# Occurrence of tocopheryl fatty acid esters in vegetables and their non-digestibility by artificial digestion juices

**DOI:** 10.1038/s41598-018-25997-2

**Published:** 2018-05-16

**Authors:** Stephanie Krauß, Vanessa Darwisch, Walter Vetter

**Affiliations:** University of Hohenheim, Institute of Food Chemistry (170b), Garbenstrasse 28, D-70599 Stuttgart, Germany

## Abstract

Tocopheryl fatty acid esters (TFAE) consist of tocopherols esterified to fatty acids, but only little is known about this substance class. In this study, twelve vegetable groups were screened on TFAE and contents of (free) tocopherols and TFAE were determined in red bell pepper, red chili pepper, cucumber and walnut (n = 5, respectively). Intact TFAE were separated by solid phase extraction from free tocopherols and analyzed by GC/MS. Highest TFAE values were determined in chili pepper (4.0–16 mg/100 g fresh weight, FW) and walnut (4.1–12 mg/100 g FW), followed by bell pepper (1.3–1.5 mg/100 g FW) and cucumber (0.06–0.2 mg/100 g FW). Contribution of TFAE to the total tocopherol content ranged from 7–84%. Neither the treatment with artificial digestion juices nor exposure to sunlight showed degradation of TFAE. This substance class might represent a hitherto overlooked storage form for free tocopherols in plants as they occur to be more stable. But as the ester bond in medium chain TFAE seems not to be fissile in the human body, they might not contribute in the same way as free tocopherols to the vitamin E activity of vegetables and might have to be determined separately.

## Introduction

**Figure 1:**
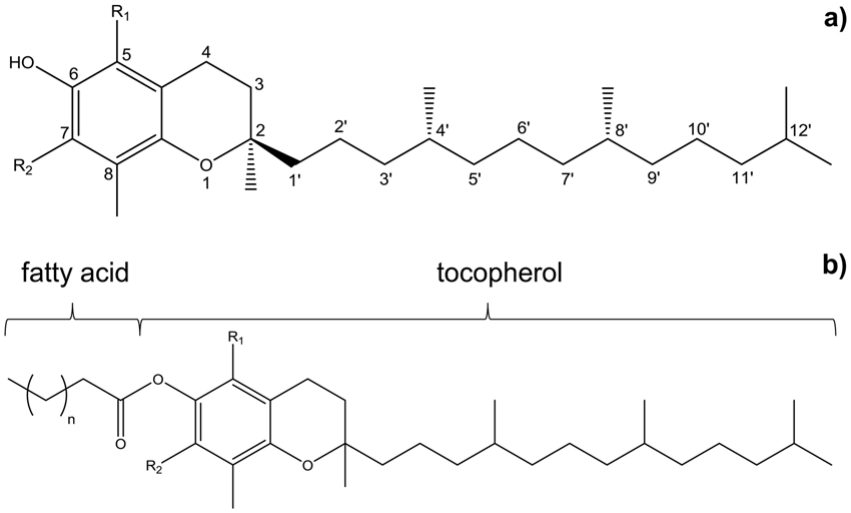
(**a**) General structure of tocopherols with: R_1_, R_2_ = CH_3_ for α-tocopherol, R_1_ = CH_3_, R_2_ = H for β-tocopherol, R_1_ = H, R_2_ = CH_3_ for γ-tocopherol and R_1_, R_2_ = H for δ-tocopherol. (**b**) Common structure of tocopheryl fatty acid esters (TFAE) with R_1_, R_2_ = CH_3_ for α-TFAE, R_1_ = CH_3_, R_2_ = H for β-TFAE, R_1_ = H, R_2_ = CH_3_ for γ-TFAE and R_1_, R_2_ = H for δ-TFAE.

Vitamin E comprises a group of fat-soluble compounds with antioxidative activity also called tocochromanols^1,2^. They differ in the number of methyl substituents on the hydrophilic 6-chromanol head group and the number of double bonds in the side chain (Fig. 1a)^2–6^. Tocopherols are potent antioxidants and function as radical scavengers^7–9^. Due to their structure tocopherols insert into the amphipathic phospholipid bilayer of cellular membranes^1,3,10,11^. Thus, they are able to protect membrane lipids as well as photosynthetic organs and seeds from oxidative stress^1,12,13^ and are widely spread in nature^1,14^. As they can only be synthesized by photosynthetic organisms and have many positive physiological impacts they are not only valuable but also essential nutrients for humans^7–9,15^. Good sources for tocopherols in general are nuts, seeds and vegetable oils^1,14^. The most abundant naturally occurring tocopherol in photosynthetic tissues is α-tocopherol^7,16^, while seeds accumulate about 10–20 times more γ-tocopherol^13^. High amounts of α-tocopherol can be found for example in wheat germ oil, sunflower oil, olive oil, hazelnuts, almonds or bell pepper whereas walnuts or pumpkin seeds are a very good source for γ-tocopherol^13,14,17,18^.

A less commonly known naturally occurring form of tocopherols is tocopheryl fatty acid esters (TFAE) in which tocopherols are esterified to fatty acids (Fig. 1b). The discovery of TFAE in the wax ester fraction of *Nymphea alba* and *Nuphar luteum* was the first time these substances were described to occur in living organisms^19^. The authors found α- and either β- or γ-TFAE in extracts of blossoms and leaves of *N. luteum* and in leaves and stems of *N. alba* with concentrations of up to 28–66 µg/g dry weight^19^. TFAE were also described to occur in the wax ester fraction of olive oil and palm oil^20^ and in the cuticular waxes of leaves of Brazil nut *(Bertholettia excelsa)* whereas α-TFAE made up about 0.2% and β-TFAE about 0.05% of the total extract^21^. TFAE also seem to be formed during the production of structured lipids as they were found there in the residues and distillates and were detected after heating of sunflower oil to 180 °C^16,22,23^. To our knowledge, nothing can be found in literature about a possible vitamin E activity of these compounds but synthesized α- and γ-tocopheryl palmitate standards from naturally isolated tocopherols were found to be nontoxic to humans^24^.

Only lately, we were able to verify the presence of TFAE in the pulp of red and yellow bell pepper together with *trans-*phytyl fatty acid esters^25^. The simultaneous occurrence of *trans-*phytyl fatty acid esters and TFAE and the fact that bell pepper contains high amounts of vitamin E^14^ indicated, that TFAE might also be a hitherto largely overlooked storage form of tocopherols when present in very high amounts.

Thus, the goal of this study was to screen more vegetables which contain high amounts of tocopherols regarding the occurrence of TFAE and to determine their composition and concentrations relative to free tocopherols. For this purpose, several α- and γ-TFAE standards were synthesized. In addition, an *in vitro*-digestion experiment was conducted which simulates the fate of such compounds in humans^26,27^. Specifically, a lipid extract containing TFAE and a standard solution was treated with artificial digestion juices. This experiment was performed to find out if the ester bond is cleaved during digestion and thus directly adds to the vitamin E activity of a vegetable or if TFAE have to be considered separately. Finally, the light stability of this substance class was investigated by exposing TFAE standard solutions to sunlight.

## Results and Discussion

### TFAE in bell pepper, chili pepper, cucumber and walnut

In SPE fraction 2, TFAE were identified by their GC/MS spectra and retention times (t_R_) in comparison to standard solutions (section *Synthesis of tocopheryl fatty acid ester standard solutions*). Miscellaneous tests were performed with different vegetables and fruits and TFAE were detected in all matrices except grapes, carrots, olives (green and black), almonds and hazelnut. Hence, the occurrence of TFAE was not directly linked to the simultaneous presence of *trans-*phytyl fatty acid esters which were detected for example in carrots and green olives. In contrast, TFAE could be detected in SPE fraction 2 of red bell pepper, chili pepper (red and yellow), rocket salad, cucumber, walnuts and maize germ oil. Different α-TFAE were detected in all samples except in walnuts and maize germ oil. In addition, γ-TFAE could be identified in all matrices and δ-TFAE only in cucumber and walnuts. Four types of vegetables (red bell pepper, red chili pepper, cucumber and walnut; n = 5) were selected for further analysis and quantification. We decided upon those vegetables as they are foods which are consumed in high amounts.

**Figure 2:**
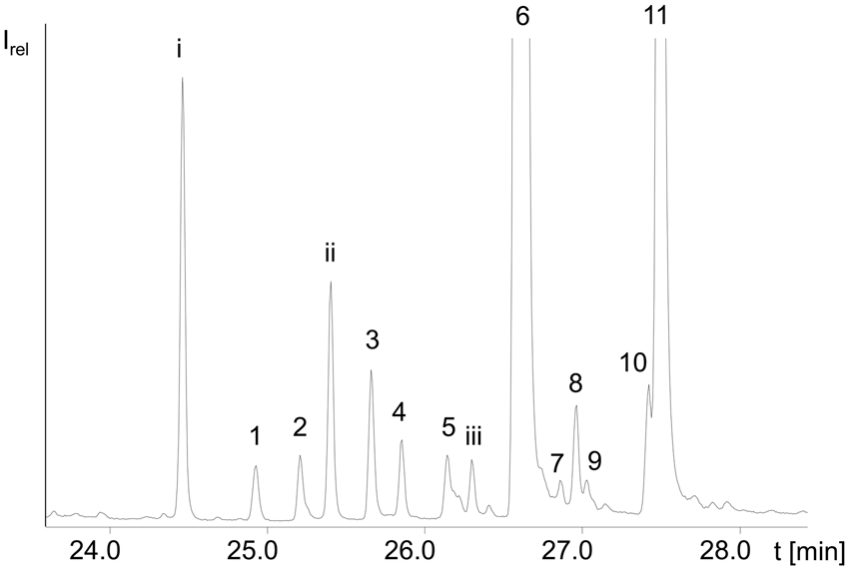
Cutout of the GC/MS-SIM chromatogram of SPE fraction 2 of a red bell pepper with i: phytyl fatty acid ester; 1: not identified; 2: co-elution of γ-TE-12:0 and assumedly β-TE-12:0; ii: phytyl fatty acid ester; 3: α-TE-12:0; 4: not identified; 5: γ-TE-14:0; iii: phytyl fatty acid ester; 6: co-elution of α-TE-14:0 and γ-TE-15:0 (ISTD-1); 7: not identified; 8: α-TE-15:0; 9: not identified; 10: tentatively identified as α-TE-16:1Δ9; 11: α-TE-16:0.

SPE fraction 2 of bell pepper (Fig. 2) featured α-TFAE mostly esterified to medium chain saturated fatty acids, i.e. α-TE-12:0 (peak no. 3), α-TE-14:0 (peak no. 6), α-TE-15:0 (peak no. 8) and α-TE-16:0 (peak no. 11). Besides, one peak (peak no. 10) eluted slightly prior to α-TE-16:0 which originated from α-TE-16:1Δ9. This was verified by saponification of fraction 2 which resulted in the detection of 16:1Δ9.

In addition, γ-TE-12:0, γ-TE-14:0 and γ-TE-16:0 were detected in red bell pepper while the peak of γ-TE-12:0 (peak no. 2) showed a small shoulder with the same fragmentation pattern as β- or γ-TFAE. As free β-tocopherol eluted only shortly before γ-tocopherol from the nonpolar stationary phase we used for the analysis of the free tocopherol fraction (see below, section *Free tocopherols in bell pepper, chili pepper, cucumber and walnut*) we assumed the same applied for β- and γ-TFAE. Saponification of the fraction verified that γ-tocopherol was predominant while β-tocopherol was present at much lower abundance. Hence, it was concluded, that peak no. 2 represented a co-elution of β- and γ-TE-12:0. Independent quantification of either γ- or β-TE-12:0 however was not possible due to the co-elution and thus the sum of both was determined.

Chili pepper showed almost the same TFAE as bell pepper which is not surprising as they both are botanically related and belong to the family of *Solanaceae*. In contrast to bell pepper, chili pepper did not contain α-TE-16:1Δ9 but an additional γ-TFAE in form of γ-TE-18:3∆9,12,15.

SPE fraction 2 of cucumber was dominated by γ-TFAE, namely γ-TE-12:0, γ-TE-14:0, γ-TE-16:0, γ-TE-18:1∆9, γ-TE-18:3∆9,12,15 and γ-TE-20:0. Additionally, the GC/MS chromatograms featured α-TE-14:0 and α-TE-16:0. Similarly to bell pepper, γ-TE-12:0 was most likely co-eluting with β-TE-12:0 (see above). GC/MS spectra of two early-eluting analytes in the chromatograms of four samples included the characteristic fragmentation pattern of δ-TFAE. Due to the lack of δ-TFAE standard solutions we could not assign a particular fatty acid moiety to those δ-TFAE. As they eluted prior to γ-TE-12:0 and γ-TE-14:0 however, they were tentatively identified as δ-TE-12:0 and δ-TE-14:0.

SPE fraction 2 of walnut differed greatly from the other samples as α-TFAE were only detected in one sample. The main difference between walnut and the other matrices was the occurrence of five abundant δ-TFAE. Additionally, γ-TE-14:0, γ-TE-15:0, γ-TE-16:0, γ-TE-18:1∆9, γ-TE-18:3∆9,12,15 and γ-TE-20:0 (only in one sample) were identified. As the δ-TFAE showed t_R_ shortly prior to γ-TFAE in the sample they could be tentatively identified as the corresponding δ-TE-12:0, δ-TE-14:0, δ-TE-16:0, δ-TE-18:0 and δ-TE-20:0.

### Free tocopherols in bell pepper, chili pepper, cucumber and walnut

Tocopherol-trimethylsilyl ethers (TMS) were identified by GC/MS data of standard solutions of α-, β-, γ- and δ-tocopherol-TMS. α-, γ- and δ-tocopherol were present in all samples, whereas β-tocopherol was found in all but walnuts.

### Concentrations of TFAE and free tocopherols

**Figure 3:**
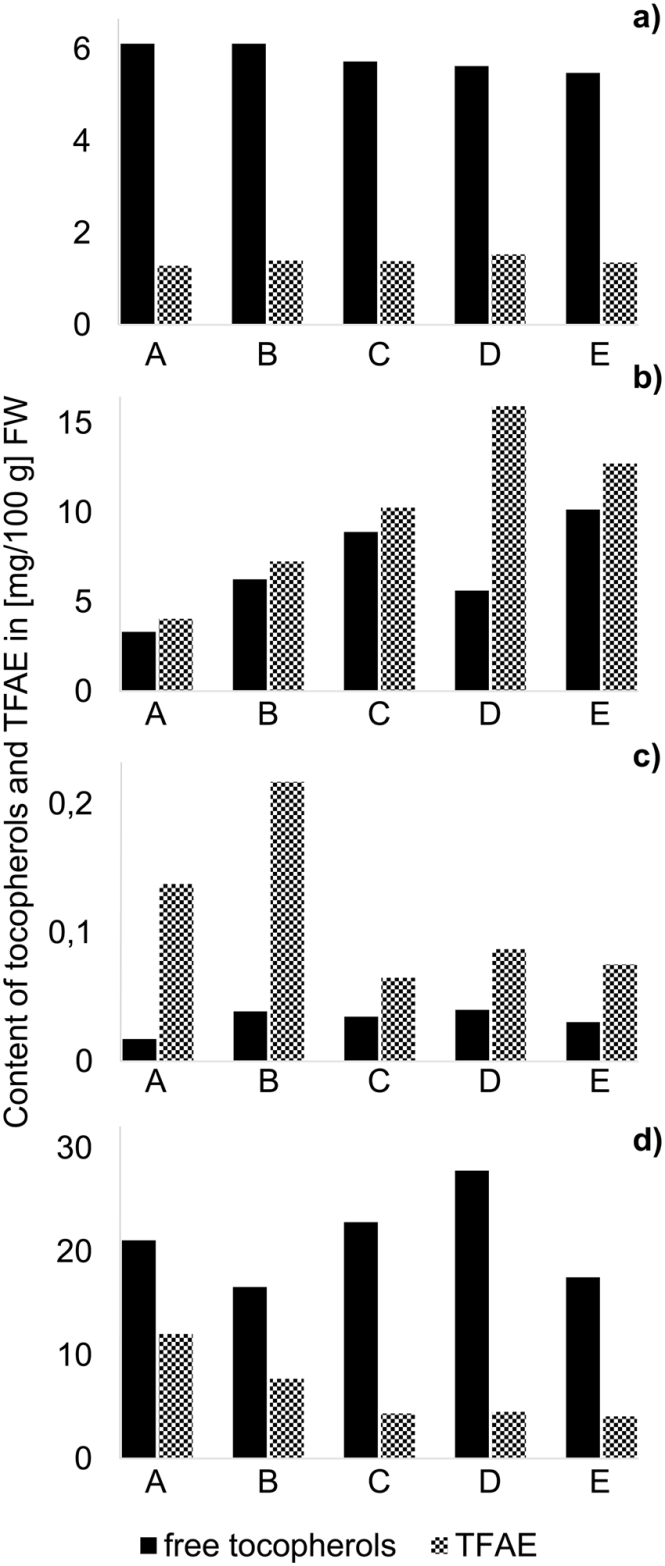
Total contents of free tocopherols and TFAE in five samples A-E of (**a**) bell pepper (**b**) chili pepper (**c**) cucumber (**d**) walnut.

**Figure 4:**
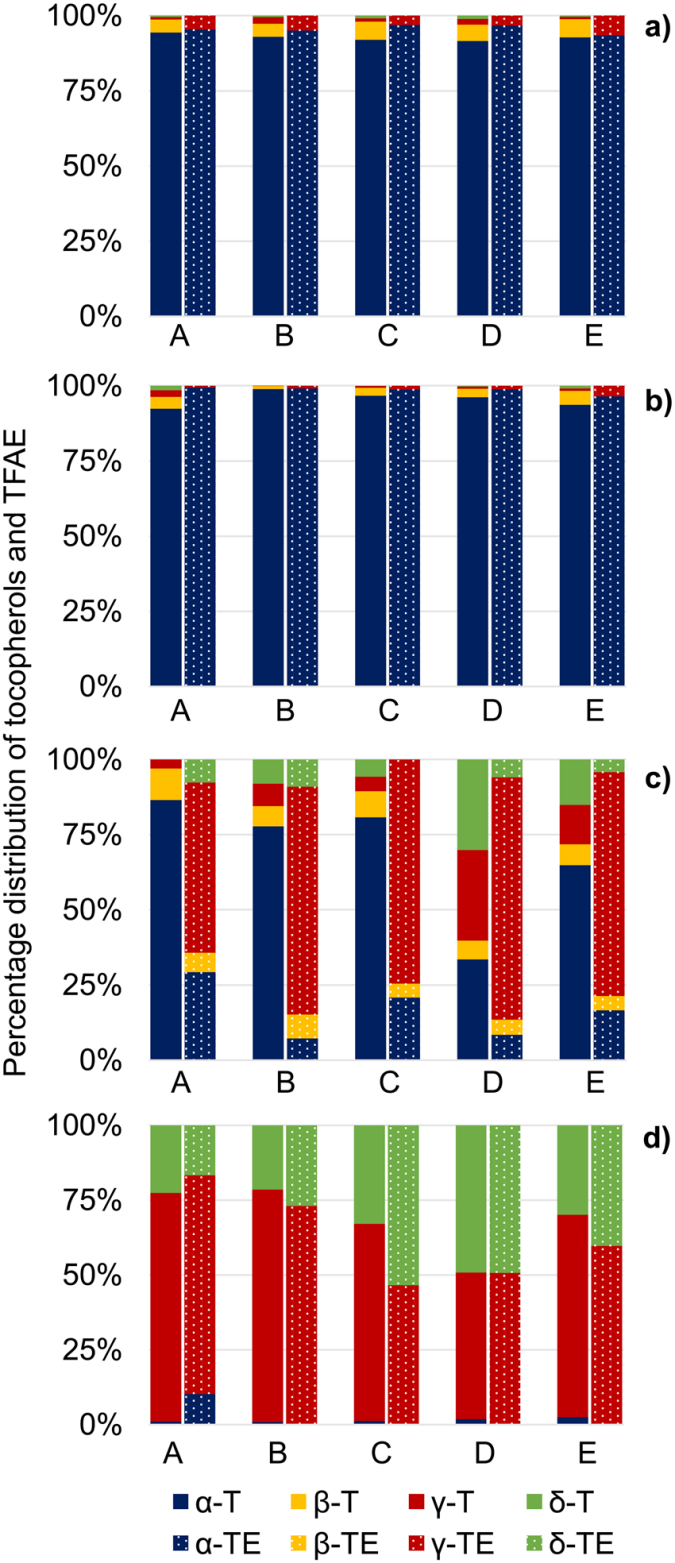
Percentage distribution of free tocopherols and TFAE in five samples A-E of (**a**) bell pepper (**b**) chili pepper (**c**) cucumber (**d**) walnut. Free tocopherols are displayed by the left bar, while TFAE are represented by the right bar of each sample. The shares of the different tocopherols and TFAE sum up to give 100%. Note, that in bell pepper and chili pepper the contribution of β-TFAE (present only in traces) could not be determined independently due to co-elutions with γ-TFAE.

TFAE contents in bell pepper ranged between 1.3–1.5 mg/100 g FW and were significantly lower (unpaired t-test, p < 0.05) than free tocopherols with the ratio of TFAE to free tocopherols (TFAE/T) being ∼0.2–0.3 (Fig. 3a). The tocopherol moiety bound in TFAE was calculated (8.2 µg (0.01 µmol) of α-TE-14:0 contained 5.5 µg (0.01 µmol) α-tocopherol) and added to the free tocopherol content. Hence, total tocopherol amounted to 6.4–7.2 mg/100 g FW with a share of ∼12–15% in form of TFAE (Table 1). In literature, total tocopherol contents of bell pepper mostly were determined after saponification^14,28–30^ and thus included free and originally esterified tocopherol. Concentrations ranged between 0.5–7.1 mg/100 g FW, in dependence of variety and stage of maturity^14,28–30^. These amounts were almost completely made up by α-tocopherol while only small amounts were ascribed to β-, γ- and δ-tocopherol^29,31,32^ which is in accordance with our results as α-tocopherol contributed with more than 92% to the contents of both free and esterified tocopherols (Fig. 4a).

**Table 1:** Mean values with standard deviations (SD, n = 5) of free tocopherols, TFAE and TFAE derived tocopherol in red bell pepper, red chili pepper, cucumber and walnut.

		Mean values with SD (n = 5) [mg/100 g FW]			
		red bell pepper	red chili pepper	cucumber	walnut
Free tocopherols	α	5.43 ± 0.31	6.59 ± 2.58	0.02 ± 0.01	0.32 ± 0.15
	β	0.31 ± 0.04	0.21 ± 0.16	0.003	n.d.
	γ	0.08 ± 0.04	0.05 ± 0.03	0.01 ± 0.01	13.89 ± 1.70
	δ	0.04 ± 0.02	0.03 ± 0.04	0.01 ± 0.01	6.95 ± 4.02
TFAE	α	1.33 ± 0.10	9.88 ± 4.53	0.02 ± 0.01	**
	β	*	*	0.01 ± 0.01	n.d.
	γ	0.06 ± 0.02	0.17 ± 0.17	0.08 ± 0.05	4.19 ± 2.97
	δ	n.d.	n.d.	0.01 ± 0.01	2.06 ± 0.26
TFAE derived tocopherol	α	0.87 ± 0.07	6.52 ± 3.00	0.01 ± 0.01	**
	β	*	*	0.01	n.d.
	γ	0.04 ± 0.01	0.11 ± 0.11	0.05 ± 0.03	2.66 ± 1.95
	δ	n.d.	n.d.	0.003	0.78 ± 0.11

*Detected in traces, but not determined independently due to co-elution; included in γ-TFAE content.

**Only in one of the walnut samples α-TE-16:0 was detected with 1.23 mg/100 g FW; share of α-tocopherol: 0.79 mg/100 g FW.

From all analyzed samples, chili pepper showed the highest TFAE contents with 4.0–16.0 mg/100 g FW (Fig. 3b). Noteworthy, TFAE dominated over free tocopherols (TFAE/T ∼1.2, Fig. 3b) and thus differed greatly from bell pepper. Sample D (TFAE/T ratio of 2.8) showed the highest proportion of TFAE (Fig. 3b). As no information about the particular variety were available, it remained unclear whether this observation could be ascribed to varietal or other differences between the chili samples. The botanical relation of chili pepper and bell pepper was reflected however, by the pattern within both the TFAE and tocopherol fraction (Fig. 4b). Literature data listed total tocopherol levels of 0.2–34.5 mg/100 g FW for different chili species^28,31,33,34^ whereas the highest amount was determined without prior saponification^33^ and thus tocopherol values could even be higher.

Cucumber had the lowest concentrations of both TFAE and free tocopherols (Fig. 3c). Noteworthy, concentrations of TFAE turned out to be significantly higher than for free tocopherols (p < 0.05) but TFAE/T for cucumber varied strongly from 1.5–6.2 (49–84% TFAE, Fig. 3c). Total tocopherol amounted to 0.08–0.11 mg/100 g FW (Table 1). Published concentrations of tocopherols in cucumber were similarly low (0.03–0.4 mg/100 g FW) and α-tocopherol was reported to be the main homologue^34–36^. Similarly, in this study free tocopherols were generally dominated by α-tocopherol. Additionally, consistently low shares of β-tocopherol (6–10%) and a constant ratio of γ- and δ-tocopherol (∼0.9) was determined except for sample A and D. Sample D stood out from all cucumbers as it contained almost equal proportions of α-, γ- and δ-tocopherol (∼30% each) (Fig. 4c). The tocopherol composition within the TFAE fraction however was similar between all cucumbers and was dominated by γ-TFAE (Fig. 4c). As no information about the variety of the particular cucumber was available, it was assumed that cucumber D could be a different species than the other samples as the tocopherol pattern was the most dissimilar. Cucumber A did not feature any δ-tocopherol. Interestingly, cucumber A was the only sample from organic cultivation which could be a possible explanation for the slightly different composition. Both assumptions cannot be verified until additional samples will be analyzed.

Walnut showed the second highest TFAE values of all matrices with 4.0–12.0 mg/100 g FW although free tocopherol contents were significantly higher (p < 0.05, TFAE/T ∼0.2–0.6) (Fig. 3d). The tocopherol composition of both fractions was dominated by the γ-homologue followed by δ-tocopherol (Fig. 4d). Free α-tocopherol was only present in trace amounts (1%) and not at all in its esterified form except for sample A (Fig. 4d). The obtained distribution of tocopherols matched the composition for walnut in literature, where also γ-tocopherol turned out to be the main homologue^18,37–39^. Total tocopherol amounted to 19.6–30.0 mg/100 g FW and thus was comparable to literature data^17,40^. As our results show, the highest contents for TFAE were determined for chili pepper and walnut, i.e. the samples which showed the highest amounts of free tocopherols. The most prominent TFAE featured the homologue which dominated in the free form (α- for chili peppers, γ- and δ-tocopherol for walnuts). These findings indicated, that TFAE might be a suitable storage form for tocopherols when they occur in very high amounts in plant tissue.

### Light stability of tocopheryl fatty acid esters

Irrespective if white or amber glass vials were used, exposure to sunlight for three days did not change the abundance of γ-TE-12:0 and γ-TE-18:3∆9,12,15 in the standard solutions. Stability of the compounds was confirmed in that no transformation products of TFAE could be detected in the GC/MS chromatograms. This indicated that TFAE could be more stable than free tocopherols which are known to be highly photosensitive^13^.

### *In vitro*-digestion experiment on the fissility of TFAE in the human body

In an *in vitro-*digestion experiment, SPE fraction 2 of a bell pepper as well as a standard solution of γ-TE-15:0 was treated with artificial digestion fluids. The GC/MS chromatogram of the digested standard solution still featured the intact TFAE. These experiments did not indicate any transformation of TFAE as free tocopherol or free pentadecanoic acid were not detected. Also, TFAE in SPE fraction 2 of bell pepper treated with digestive enzymes were still intact and free of (free) tocopherols. Besides TFAE, SPE fraction 2 also contained *trans-*phytyl fatty acid esters. It was recently shown by Krauß *et al*.^27^ that these substances were cleaved by the lipase also used in this experiment. In contrast to TFAE, the *trans-*phytyl fatty acid esters were cleaved during the digestive process. This finding verified that the enzymes used in the experiment were active and outlined the stability of TFAE towards digestive enzymes.

Tocopherols used as nutrition supplements often are given as tocopheryl acetates as they are more stable towards oxidation than their free form^41–45^. Studies showed that hydrolysis of α-tocopheryl acetate occurred in the intestine^41,42,44^ but still the ester had a lower bioavailability than the free form^43,45^. Interestingly, the more hydrophilic the acid moiety the lower was the absorption of the ester, as was the case for α-tocopheryl succinate and nicotinate^45^. The results presented in the present study lead to the assumption that also the chain length of the acid moiety has influence on the fissility of tocopheryl esters as γ-TE-15:0 remained intact after treatment with digestive fluids. Therefore, an additional *in vitro*-digestion experiment was conducted with α- and γ-tocopheryl acetate which resulted in cleavage of the ester bond as GC/MS chromatograms of the solutions featured free α- and γ-tocopherol after digestion.

Based on these results it was concluded, that TFAE (in contrast to tocopheryl acetates) are non-fissile in the human body at least when esterified to medium chain fatty acids. These findings also raised the question if and to what extent TFAE show vitamin E activity. Hence, they might have to be determined separately regarding the vitamin E content and activity of foods.

Further investigations regarding the absorption and vitamin E activity of TFAE as well as the influence of botanical variances and different cultivation practices on the TFAE composition will have to be carried out.

## Methods

### Samples

Edible parts of red bell pepper, red chili pepper, cucumber, grapes, olives (green and black), rocket salad, carrots, walnuts, hazelnuts, almonds, maize-germ oil and linseed oil were analyzed on TFAE. The first matrices (red bell pepper to olives) were chosen for analysis as we detected *trans*-phytyl fatty acid esters in their lipid extracts during another study^27^ while the remaining samples were randomly chosen due to their known vitamin E content^9,13,35^. Samples were purchased between April and June 2017 in local supermarkets in Stuttgart, Germany, except one of the walnut samples (D) which originated from private cultivation (Allfeld, Baden-Württemberg, Germany). The remaining walnut samples were from California (A, B, E) and from Chile (C). Bell peppers originated from Spain (A-D) and the Netherlands (E), chili peppers also were from Spain (A, B, D), from Morocco (C) and from Sardinia (F), while cucumbers originated from Spain (A, B) and from Germany (C-E). As no information regarding the varieties of the samples were given by the product labels and no photos were taken during sample preparation which could hint to a specific variety, there may occur variations in attempts to repeat the results reported in this manuscript – however, this will hopefully not affect the major conclusions.

### Chemicals

Chemicals used were identical to Krauß *et al*.^25,27^ with few exceptions. (*R,R,R*)-α-tocopherol (≥97%) and (*R,R,R*)-γ-tocopherol were purchased from Sigma-Aldrich (Steinheim, Germany), while β-tocopherol and δ-tocopherol were from Supelco Analytical (Bellefonte, USA). Thionyl chloride, myristic acid (≥98%) and α-linolenic acid were from Fluka (Steinheim, Germany) and lauric acid and pentadecanoic acid (both >99%) were ordered from Merck (Darmstadt, Germany).

### Synthesis of tocopheryl fatty acid ester standard solutions

Chemical esterification of α- and γ-tocopherol with fatty acids was carried out according to the method of Lusby *et al*. suggested for steryl esters^46^ with toluene being used as solvent instead of benzene. About 0.5 mmol of the particular fatty acid was dissolved in 10 mL of toluene and was heated under reflux for 4 h after adding about 1.5 mmol of thionyl chloride. Excessive thionyl chloride was removed by rotary evaporation and the volume of the solution was reduced to about 2 mL. Then, 0.4 mmol of α- or γ-tocopherol and 10 mL of toluene were added and the solution was stirred for another 48 h at room temperature. Non-esterified α- or γ-tocopherol and excessive free fatty acids were separated from TFAE by solid phase extraction (SPE) according to Krauß *et al*.^25^ on 5 g of deactivated silica gel (20% with water). TFAE were separately eluted with 30 mL of *n*-hexane/ethyl acetate (99:1, *v/v*). The solvent was removed by rotary evaporation and the residue was determined gravimetrically and re-diluted in 1 mL of *n*-hexane. α- and γ- tocopherol were each esterified with lauric acid, myristic acid, pentadecanoic acid, palmitic acid, stearic acid, oleic acid, linoleic acid, α-linolenic acid and arachidic acid to give the corresponding TFAE.

### Sample preparation

#### Isolation of TFAE and free tocopherols

Lipid extraction and sample clean-up was carried out in accordance with Krauß *et al*.^25,47^. In short, 4–7 g of homogenized sample material (for walnut 0.2 g) were extracted by focused open-vessel microwave assisted extraction according to Batista *et al*.^47^ with the azeotropic mixture of cyclohexane/ethyl acetate (46:54, *w/w*) and the volume of the lipid extracts was adjusted to 4 mL. Separation of lipid classes was obtained by SPE on deactivated silica gel (20%, with water). Two milliliters of each lipid extract, supplemented with 10 µg of α-TE-18:3∆9,12,15 (ISTD-1; in case of bell pepper 10 µg of γ-TE-15:0 was used as ISTD-1), was placed on the SPE column and elution was carried out with four different mixtures of *n*-hexane/ethyl acetate. TFAE eluted into SPE fraction 2 (*n*-hexane/ethyl acetate, 99:1, *v/v*), while free tocopherols were separated into SPE fraction 3 (*n*-hexane/ethyl acetate, 95:5, *v/v*). The volume of SPE fraction 2 was adjusted to 1 mL of *n*-hexane and after 3.5 µg of 5α-cholestane was added (ISTD-2, standard to level out differences in injection volume) was introduced to GC/MS for the measurement of intact TFAE. For cucumber extracts a concentration factor of 5 was chosen while for the other samples no concentration was necessary. An aliquot of SPE fraction 3 corresponding to about 50 µg was trimethylsilylated according to Hammann *et al*.^48^ and 3.5 µg of ISTD-2 was added before analysis with GC/MS.

#### Light stability of tocopheryl fatty acid esters

Solutions of 10 µg/mL of γ-TE-12:0 and of γ-TE-18:3∆9,12,15 were exposed to sunlight in white glass as well as in sealed amber glass 1.5 mL-vials for three days. Twice a day aliquots of 160 µL were taken, transferred to amber glass vials and introduced to GC/MS analysis after derivatization to trimethylsilyl ethers and addition of 3.5 µg of ISTD-2.

#### *In vitro-*digestion of tocopheryl fatty acid esters

*In vitro*-digestion experiments with TFAE were carried out in accordance with Heinlein *et al*.^26^ with the same concentrations of the digestion fluids and the same procedure as was used by Krauß *et al*.^27^. For the experiment an aliquot of SPE fraction 2 of a bell pepper sample as well as a standard solution of α-TE-15:0 (∼0.3 mg, respectively) was used and treated with the digestion juices. As control sample, the standard solution was only treated with water instead of digestion fluids. After the digestion process the solutions were acidified, extracted three times with 10 mL of *n*-hexane and the volume was adjusted to 1 mL. After adding 3.5 µg of ISTD-2 the samples were measured by GC/MS.

#### Analysis with GC/MS

Intact TFAE and the samples of the *in vitro*-digestion experiment were analyzed with a 6890/5973 GC/MSD system (Agilent Technologies, Santa Clara, USA) equipped with an on-column injection system using the parameters of Krauß *et al*.^25^. Data were recorded in full scan mode (*m/z* 50–800) and in selected ion monitoring (SIM) mode using the following SIM ions with a dwell time of 30 ms each: *m/z* 430, *m/z* 416 and *m/z* 402 represents the base peak for α-TFAE, β-/γ-TFAE and δ-TFAE, respectively. It is formed by loss of the fatty acid moiety with H-transfer from the α-C-atom to the oxygen atom included in the ester bond at the tocopherol^19,21,22^. Cleavage of the side chain by a retro-Diels-Alder reaction leads to a second abundant fragment ion at *m/z* 165 (α-TFAE), *m/z* 151 (β-, γ-TFAE) or *m/z* 137 (δ-TFAE). Additionally, *m/z* 502 and *m/z* 488 for α-tocopheryl- and γ-tocopheryl-trimethylsilyl ether (TMS) and *m/z* 217 and *m/z* 353 for 5α-cholestane (ISTD-2) were included. Electron multiplier voltage in SIM mode was increased by 500 V relative to full scan mode. As in most cases the low abundant molecular ion M^+^ (~2–4% of the base peak)^19^ of TFAE could not be detected in sample chromatograms, specific TFAE were identified by t_R_ in comparison to synthesized standards (see section *Synthesis of tocopheryl fatty acid ester standard solutions*).

Quantification was carried out via ISTD-1 after correcting the peak areas of the quantification ion (*m/z* 430, *m/z* 416, *m/z* 402 for α-, β-/γ-and δ-TFAE, respectively) by the individual SIM-responses for α- and γ-TFAE. As β-TFAE show the same fragmentation pattern as γ-TFAE, the peak areas of β-TFAE were corrected by the SIM-response of γ-TFAE while peaks identified as δ-TFAE were corrected by the SIM-response of α-TFAE as those two species are structurally more similar to each other than γ- and δ-TFAE. The limit of detection (LOD) and limit of quantification (LOQ) ranged for α-TFAE between 1–180 µg/100 g FW and 1.6–270 µg/100 g FW, respectively and for γ-TFAE between 0.14–23.8 µg/100 g FW and 0.24–0.40 µg/100 g FW, depending on the analyzed vegetable. The smallest values for LOD and LOQ were obtained for cucumber and the highest for walnuts.

TMS ethers of free tocopherols were analyzed with another 6890/5973 N MSD system equipped with a split/splitless injection system (Agilent Technologies, Santa Clara, USA) described in detail by Krauß *et al*.^25^. Sample solutions (1.0 µL) were injected in splitless mode and the injector was heated to 250 °C. After 1 min at 55 °C, the oven temperature was raised to 255 °C by 20 °C/min, then with a rate of 1.5 °C/min to 283 °C and at 15 °C/min to the final temperature of 300 °C which was held for 5 min. Data were collected after a solvent delay of 7.0 min in full scan mode (*m/z* 50–650) and in SIM mode using *m/z* 209 and *m/z* 474 for δ-tocopheryl-TMS, *m/z* 223 and *m/z* 488 for β-/γ-tocopheryl-TMS, *m/z* 237 and *m/z* 502 for α-tocopheryl-TMS and *m/z* 217 and *m/z* 357 for 5α-cholestane (ISTD-2) throughout the run with a dwell time of 35 ms each.

Silylated tocopherols were quantified via an external standard mixture of α-, β-, γ- and δ-tocopherol (5 ng/µL each) after correcting the peak areas of the quantification ion (*m/z* 502, *m/z* 488, *m/z* 474 for α-, β-/γ- and δ-tocopherol-TMS, respectively) out of the GC/MS-SIM chromatograms by their individual SIM-responses. LOD and LOQ was 0.06–9.7 µg/100 g FW and 0.17–27.6 µg/100 g FW for α-tocopherol, 0.05–8.4 µg/100 g FW and 0.2–34.3 µg/100 g FW for β-tocopherol, 0.03–4.5 µg/100 g FW and 0.09–14.9 µg/100 g FW for γ-tocopherol and 0.04–5.84 µg/100 g FW and 0.09–14.4 µg/100 g FW for δ-tocopherol, depending on the vegetable analyzed. Lowest LOD and LOQ were determined for cucumber and highest for walnuts.

### Data availability

All data generated or analyzed during this study are included in this published article.
